# Autophagy and Apoptosis in Rabies Virus Replication

**DOI:** 10.3390/cells13020183

**Published:** 2024-01-18

**Authors:** Saisai Li, Bowen Xu, Yongwen Luo, Jun Luo, Shile Huang, Xiaofeng Guo

**Affiliations:** 1 College of Veterinary Medicine, South China Agricultural University, Guangzhou 510642, China; 20222028021@stu.scau.edu.cn (S.L.); ywluo@scau.edu.cn (Y.L.); 2 College of Veterinary Medicine, Henan Agricultural University, Zhengzhou 450046, China; xubowen600@outlook.com; 3 Department of Biochemistry and Molecular Biology, Louisiana State University Health Sciences Center, 1501 Kings Highway, Shreveport, LA 71130-3932, USA; shile.huang@lsuhs.edu; 4 Department of Hematology and Oncology, Louisiana State University Health Sciences Center, Shreveport, LA 71130-3932, USA; 5 Feist-Weiller Cancer Center, Louisiana State University Health Sciences Center, 1501 Kings Highway, Shreveport, LA 71130-3932, USA

**Keywords:** rabies virus, autophagy, apoptosis, virus replication

## Abstract

Rabies virus (RABV) is a single-stranded negative-sense RNA virus belonging to the *Rhabdoviridae* family and *Lyssavirus* genus, which is highly neurotropic and can infect almost all warm-blooded animals, including humans. Autophagy and apoptosis are two evolutionarily conserved and genetically regulated processes that maintain cellular and organismal homeostasis, respectively. Autophagy recycles unnecessary or dysfunctional intracellular organelles and molecules in a cell, whereas apoptosis eliminates damaged or unwanted cells in an organism. Studies have shown that RABV can induce both autophagy and apoptosis in target cells. To advance our understanding of pathogenesis of rabies, this paper reviews the molecular mechanisms of autophagy and apoptosis induced by RABV and the effects of the two cellular events on RABV replication.

## 1. Introduction

Rabies is a highly fatal zoonotic disease that seriously affects the health of humans and animals worldwide. Rabies virus (RABV) belongs to the *Rhabdoviridae* family and *Lyssavirus* genus, and is a single-stranded negative-sense RNA virus with a genome length of approximately 12 kb [1] encoding five proteins: nucleoprotein (N), phosphoprotein (P), matrix protein (M), glycoprotein (G), and RNA polymerase (L) [2]. RABV primarily infects neurons, but there is evidence that it can also infect astrocytes and microglia [3]. RABV can cause fatal viral encephalitis in various host species. Although vaccination and immune globulin can prevent the development of rabies after exposure, there are no effective drugs to treat rabies [4]. The high mortality rate of rabies poses a serious threat to human health and public safety [5]. Therefore, it is of great importance to study the replication and pathogenic mechanisms of RABV.

Each protein of RABV plays a critical role in its replication and immune escape [6,7]. The RABV N protein wraps the RNA genome, forming a tightly wrapped N-RNA complex known as ribonucleoprotein (RNP) [4]. RNP together with the L and P proteins form a helical nucleocapsid (NC), which serves as a template for RABV RNA transcription and replication [8]. The RABV P protein is a catalytic cofactor of polymerase L and can disrupt host interferon-mediated antiviral responses [9]. The RABV M protein, which surrounds the NC and is located between the N and G proteins, interacts with the intramembrane structural domains of the G protein and RNP, participates in virus budding and RNA replication, and connects NC with the virus envelope [5]. The RABV G protein is a trimer and is the only protein exposed on the rhabdovirus membrane surface [10,11]. Its extracellular region is related to cell receptor recognition, adhesion, and viral invasion [11,12]. The G protein has also been shown to be closely related to neuronal apoptosis [13]. In the early stage of RABV infection, the virus is transported through the neuromuscular junction reversely along the axons to the central nervous system [14].

Autophagy is an evolutionarily conserved and genetically regulated process, which maintains cellular homeostasis [15]. Autophagy is divided into macroautophagy, microautophagy, and chaperone-mediated autophagy [16,17,18]. During autophagy, the formation of autophagosomes and double-membrane vesicles is crucial [19]. They are formed by sequestering cytosolic materials into an expanding membrane sac, the phagophore. Once matured, autophagosomes fuse with lysosomes, where the cargo-containing membrane compartment is lysed, and the contents are degraded in the cell [17,18,20]. Subsequently, the sugars, lipids, amino acids, nucleotides, and other nutrients produced by degradation are fed back to the cell for reuse [19,21]. Many studies have shown that there are complex interactions between the viral infection process and autophagy [21]. Autophagy has a dual role. On the one hand, upon virus infection, host cells activate autophagy, generating a series of defense mechanisms to clear viral particles in the cells; on the other hand, viruses can hijack autophagosomes, forming a membrane-bound protective environment to provide metabolites and energy for their self-replication [22,23,24]. Studies have shown that most negative-sense RNA viruses [25,26], including RABV, can induce autophagy during infection, but the degrees of autophagy induced are dependent on RABV strains and the host cell lines used [25,27].

Apoptosis is also an evolutionarily conserved and genetically regulated process, which eliminates damaged or unwanted cells maintaining organismal homeostasis [28,29]. Apoptosis occurs through two pathways: the extrinsic and intrinsic apoptotic pathways [30,31]. Previous studies have shown that RABV may induce two mechanisms of apoptosis: immune-mediated death, mediated by the interaction of caspase-1 and interleukin-1 (IL-1) activating death receptor ligands, and enzyme-mediated death, including lysosomal protease and calcium-dependent neutral protease [32]. However, RABV strains, inoculation methods, and host cell types can induce different types of apoptosis [27].

During the RABV infection process, how does the virus induce autophagy? Which apoptotic pathways are activated? What is the relationship between autophagy and apoptosis? Does autophagy inhibit or promote virus replication? To address these questions, this paper will review the related literature and discuss the mechanisms of autophagy and apoptosis caused by RABV and the effects of the two cellular events on RABV replication.

## 2. Overview of Autophagy

Autophagy is a highly conserved process that constantly occurs in most eukaryotic cells [5,33]. It plays a crucial role in clearing misfolded proteins, malfunctioning organelles, and in the recycling and reuse of energy and nutrients [34,35]. Especially in nerve cells, dysfunction of the dynamic equilibrium mechanism can lead to the accumulation of misfolded proteins, which, in turn, can result in neurodegeneration [34], leading to neurodegenerative diseases [20,36]. Various conditions, such as energy stress, heat shock, hypoxia, excessive reactive oxygen species and reactive nitrogen species, organellar dysfunction, and microbial infections, can trigger autophagy [37]. Autophagy is a “double-edged sword”. On the one hand, autophagy is known as a protective mechanism in the body, which can specifically clear the aforementioned cell-toxic substances and maintain intracellular homeostasis [5,36]. On the other hand, autophagy may result in cell death under certain conditions, especially severe cellular stress [38]. Autophagy of host cells caused by virus invasion is one of the forms of heterogeneous autophagy, in addition to foreign pathogens such as bacteria and fungi. Xenogeneic autophagy is a kind of selective autophagy, in which foreign pathogens are specifically bound to the target sites by selective receptors and degraded and cleared by autophagy [39,40].

### 2.1. General Mechanisms of Autophagy

Autophagy is a complex biological process, which involves multiple steps, including autophagosome initiation, nucleation, amplification and elongation of the autophagosome membrane, and closure and fusion with lysosomes, ending with the degradation of products within the vesicle [41]. Since most of autophagy induced by rabies virus infection occurs in mammals, here we focus on discussing the general mechanisms of autophagy in mammalian cells, whereas heterologous autophagy induced by rabies virus invasion will be described in detail in Section 2.4. The occurrence of autophagy is mainly dependent on three proteasomal complexes, which are the ULK1 complex, the PI3KC3 complex, and the ATG16L1 complex; furthermore, these three complexes are able to sequentially regulate the process of autophagy [42]. Each complex consists of multiple components, and the ULK1 complex includes ULK1, ULK2, ULK3, as well as ATG101 and RB1CC1/FIP200 [43,44]; PI3KC3 complexes include BECN1, PIK3C3/Vps34, PIK3R4/P150, NRBF2, and ATG14 or UVRA [44]; the ATG16L1 complex includes ATG12, ATG5, and ATG16L1 [41]. We will present the functions performed by each complex in turn, based on the sequence of steps in which autophagy occurs. Firstly, the process of autophagosome initiation is closely linked to the ULK1 complex [43], which constitutes the carrier of phagosomes and activates the class III phosphatidylinositol 3-kinase (PI3KC3) complex to nucleate the phagocytosis vector [45]. Under ATP-rich conditions, the mammalian target of rapamycin complex 1 (mTORC1) stimulates the phosphorylation of ULK1 and inactivates the ULK1 complex, inhibiting the initiation and onset of autophagy. In contrast, when ATP levels are decreased, AMPK is activated, which inhibits mTORC1 through phosphorylation of TSC2 and Raptor; this inhibition attenuates the phosphorylation of ULK1 by mTORC1, leading to the interaction of AMPK with ULK1, which stimulates the formation of autophagosomes [46,47,48]. Notably, mTOR and AMPK do not phosphorylate ULK1 at the same sites, and even act antagonistically [48]. The ULK1 kinase complex has been shown to be a central node for inputs from AMPK and mTORC1, and it has been specifically noted that ULK1 can be activated directly by AMPK phosphorylation without needing to inhibit mTORC1 [48]. ULK1 can be directly activated by AMPK phosphorylation in vitro, and the phosphorylation of the S317 and S777 sites is essential for AMPK activation of ULK1 in vivo and in vitro [37,48]. Interestingly, during selective autophagy, the ULK1 complex can be activated through autophosphorylation by the accumulation of ubiquitinated targets [37,45]. That is, ULK1 can be activated independently of the regulation of upstream factors, an observation that has been demonstrated in mitochondrial autophagy [37,45,49]. Secondly, PI3KC3 is phosphorylated and activated by the ULK1 complex, which is involved in autophagosome nucleation. The main mechanism is that after activation of the Beclin1-VPS34 complex by the ULK1 complex, VPS34 is able to generate phosphatidylinositol-3-phosphate-rich regions on membrane donors, and phosphatidylinositol-3-phosphate (PtdIns3P) recruits WIPI1-4 and DFCP1 [50]. At this point, the initial carrier of the autophagosome is basically formed, followed by the membrane expansion and closure of the autophagosome. The process of membrane expansion involves the formation of the ATG16L1 complex and the lipidation of LC3. The formation of the ATG16L1 complex involves multiple ubiquitin-like coupling processes [50]. First, ATG7 and ATG10 participate in the coupling process of ATG12 and ATG5 as E1, E2 enzymes to form the first complex, which in turn binds to ATG16L1 to form the second complex, also known as the ATG16L1 complex, and acts as an E3 enzyme to stimulate LC3 lipidation [44,49,50,51]. There are many kinds of ATG8 family proteins; here, we will only talk about LC3. LC3-I, which undergoes ATG4B cleavage of the C-terminal arginine, is able to covalently bind to phosphatidylethanolamine (PE) on the autophagosome membrane and lipolyze to form LC3-II in the presence of ATG7 and ATG3 [51]. Finally, ATG16L1 binds LC3-II to the membrane of autophagy carriers by interacting with WIPI2 [52]. By repeating this, the membrane can be gradually lengthened. In nascent autophagic vesicles, the ends of the circular double membrane undergo closure by ESCRT-III, forming a complete autophagic vesicle [53,54]. In mammals, autophagic vesicles usually fuse with lysosomes to form autophagic lysosomes. Finally, the engulfed cargo is degraded by enzymatic digestion [53,54].

### 2.2. Complex Interactions between Autophagy and Viruses 

A large body of evidence suggests that there is a complex interaction between autophagy and invading viruses, and that autophagy has a dual role in viral infections [35,46]. Most viruses recognize pathogen-associated molecular patterns (PAMPs) via pattern recognition receptors (PRRs) during infection of host cells; in mammals, the endosomal TOLL-like receptor is predominant, which stimulates downstream junctions to interact with BECN1, activating autophagy [50,55]. Autophagy, as a host cell antiviral defense mechanism, induces the production of interferon and initiates innate immunity, and in the later stages of viral infection, autophagy can selectively degrade the immune components associated with viral particles and deliver antigens to T-lymphocytes to aid in the elimination of pathogens [50,56]. In turn, viruses have evolved a variety of escape strategies to resist cellular autophagy; they can inhibit autophagosome initiation, block autophagosome maturation, disrupt the process of fusion of autophagosomes and lysosomes to evade autophagosome recognition, and even make use of the membrane structure of the autophagosome to provide a site for their own replication [57]. Autophagosomes can serve as relevant replication scaffolds for certain cytoplasmic RNA viruses. The M2 protein of influenza virus, the NEF protein of HIV, and the P protein of RABV all target BECN1, completely inhibit autophagy or block autophagic flux, and, thus, escape autophagy [58,59,60]. In sum, autophagy has either pro-viral or anti-viral functions depending on the virus and the viral replication cycle.

### 2.3. Induction of Autophagy by RABV

Rabies virus has been shown to cause autophagy in neuronal cells during infection [61]. The accumulation of autophagic vesicles is always observed during RABV infection, and there are two mechanisms regarding the accumulation of autophagic vesicles. One is the increased synthesis rate of autophagosomes, unchanged degradation rate, and increased autophagic flux; the other is an unchanged synthesis rate of autophagosomes, but an impaired lysosomal degradation process and decreased autophagic flux [62]. Thus, RABV causes complete autophagy if it does not affect the fusion and degradation process of autophagic lysosomes during replication and, conversely, triggers incomplete autophagy. Peng et al. demonstrated that the wild-type RABV GD-SH-01 strain triggered complete autophagy in SK cells; incomplete autophagy was triggered in NA cells under the same multiplicity of infections (MOI) and it was found that the weakening strain HEP-Flury caused less pronounced autophagy in the cells as compared to the strong strain [27]. This demonstrates that autophagy induced by RABV is not only related to the strength of the virulent strain, but also to the MOI of the host cell and the viral infection. However, in another study, both the strong strain CVS-11 and the weak strain HEP-Flury induced incomplete autophagy in NA cells [58]. Whether the debilitating strain HEP-Flury can cause incomplete autophagy in NA cells or not is controversial. This is probably because of the differences in the strains and cells used in each laboratory. In addition to this, recent studies have shown that CVS-11 and HEP-Flury are also able to induce incomplete autophagy in BV2 (microglia) and the degree of autophagy is positively correlated with the dose of the receiving agent [63]. Overall, there is more evidence pointing to the ability of RABV to cause incomplete autophagy in cells, with the exception of SK cells. 

### 2.4. Regulation of Autophagy-Related Pathways by RABV

The mechanism of rabies virus-induced viral autophagy is still not well understood. Here, we discuss the results of previous studies with the aim of clarifying the mechanism of virus-induced autophagy. We analyze one strategy for the regulation of autophagy by RABV virus in terms of its need for replication. First, RABV requires the site and environment of autophagosomes to help it replicate and escape the immune system, so RABV infection is usually able to activate autophagy [57,61]. RABV targets a number of autophagy-activated targets for regulation, such as mTOR, AKT, AMPK, MAPK (ERK1/2), ULK1, CASP2, and others. Second, in order for RABV to proliferate further in large numbers, it usually causes only the onset of autophagy and the accumulation of autophagosomes but prevents the fusion of autophagosomes and lysosomes [61,64,65,66]. For example, the P protein of RABV can bind Beclin1 in the PI3KC3 complex and hinder the formation of autophagic lysosomes. In conclusion, RABV can regulate the process of cellular autophagy according to its own needs [67,68,69].

#### 2.4.1. RABV Causes Autophagy by Inhibiting mTOR with AMPK

In rabies virus-induced complete and incomplete autophagy, mTOR is inhibited through different signaling pathways [58,70,71]. In the study by Peng et al., the RABV wild strain GD-SH-01 was able to induce complete autophagy in SK cells and the AMPK signaling pathway was found to be activated. The phosphorylation of mTOR and its downstream target RPS6KB was further inhibited to activate autophagy [27]. Liu et al. found that AMPK-AKT-mTOR and AMPK-MAPK signaling pathways were activated in incomplete autophagy induced by RABV infection [58,67]. AMPK is located upstream and regulates the initiation of autophagy, and AMPK positively regulates ULK1 and negatively regulates mTOR, leading to autophagy [58,72].

#### 2.4.2. RABV N/P-Induced Autophagy by Downregulating CASP2

Tiwari et al. demonstrated that CASP2 not only regulates the apoptotic process, but also plays an important role as a negative regulator of autophagy [73]. Deletion of CASP2 induces downregulation of the mTOR pathway and upregulation of AMPK activation in autophagy [58]. This suggests that RABV infection downregulates CASP2, and that CASP2 deletion further inhibits mTOR and activates AMPK; thus, the end result is that AMPK activates the ULK1 complex to enable the initiation of autophagy, favoring RABV replication. The RABV N/P proteins play a very important role in the CASP2 pathway [58]. Both *N* and *P* genes can cause upregulation of LC3-II levels, but only the P protein shows a dose-dependent effect. Both viral proteins N and P can reduce CASP2 phosphorylation levels and induce autophagy [58,74].

#### 2.4.3. RABV P-Induced Autophagy by Binding to BECN1

The BECN1 protein, encoded by the *BECN1* gene, is a mammalian ortholog of yeast ATG6 [69]. BECN1, as a scaffolding protein, interacts with Vps34 and Vps15, together with either ATG14L or UVRAG, to, respectively, form two Class III PI3K complexes, Complex 1 (C1) and Complex 2 (C2), which regulate autophagy and/or membrane trafficking [75]. The anti-apoptotic proteins Bcl-2 and Bcl-xL can bind to BECN1 and inhibit its function [76]. When this binding is disrupted, BECN1 is activated, leading to autophagy. It has been shown that the P protein of RABV interacts with BECN1, which results in a decreased expression of CASP2 and incomplete autophagy by activating the BECN1-CASP2-AMPK-AKT-mTOR and BECN1-CASP2-AMPK-MAPK signaling pathways [58]. Further studies showed that the RABV P protein contains five splice variants, namely, the full-length phosphorylated protein P1 and the four phosphorylated protein truncates P2-P5. Among them, the P5 protein is mainly located in the nucleus and triggers incomplete autophagy by directly binding to the N-terminal loop structure of BECN1 [58,67]. The P5 protein wraps immature autophagic vesicles in a circular structure to prevent the fusion of autophagosomes and lysosomes, thereby promoting RABV self-replication [67]. In addition, a recent study has shown that Trim25 can interact with CVS-P protein through the coiled-coil domain (CCD) and disrupt the stability of the P protein by inducing complete autophagy, thereby inhibiting RABV replication [77].

#### 2.4.4. IFITM3 Inhibition of Autophagy by Inhibiting mTORC1 and ULK1

It is well known that RABV can hijack the autophagy process to help its own replication. Studies have shown that IFITM3 (interferon-induced transmembrane protein 3) inhibits RABV replication by inhibiting mTORC1/ULK1-dependent autophagy in RABV-infected cells [64]. However, the content of autophagy substrate SQSTM1/p62 does not change significantly. It has been confirmed that the type of autophagy induced by IFITM3 by inhibiting mTORC1 and ULK1 is incomplete autophagy [78,79]. Overexpression of IFITM3 increases the phosphorylation level of mTORC1, and activated mTORC1 can further phosphorylate ULK1 (Ser757) and inhibit its function, thereby terminating the initiation of autophagy [78,80].

#### 2.4.5. Effect of Autophagy on RABV Proliferation

Autophagy is involved in RABV infection and promotes its replication, but this is apparently dependent on RABV strains and host cell lines. For instance, in NA cells infected with two RABV strains (pathogenic CVS-11 strain and inactivated SRV9 strain), the protein expression levels of LC3 are not altered at 12 h post-infection (hpi), but increase at 24 hpi in both RABV-infected groups; at 36 or 48 hpi, the LC3-II levels in the cells infected with CVS-11 but not SRV9 decrease to those found in the mock-infected cells [27,81]. The autophagy caused by wild-type RABV GD-SH-01 is cell line dependent, but in cells with autophagy, regardless of complete or incomplete autophagy, the replication and transcription levels of RABV are higher than those in the control group [58]. In addition, the titer of RABV is significantly increased by autophagy induced by rapamycin [58]. On the contrary, the replication level of RABV is decreased after the inhibition of autophagy with 3-MA [58]. The above observations support the idea that autophagy promotes RABV replication.

The decrease in autophagy flux and the accumulation of autophagosomes are beneficial to the replication of RABV. It has been confirmed that the virulent strain CVS-11 can induce incomplete autophagy in N2a cells and promote the accumulation of autophagosomes [82]. SQSTM1/p62 protein, a marker of autophagy execution, is accumulated in GD-SH-01-infected NA cells with an increase in infection time, while the expression level of BECN1, a protein critical for autophagosome formation, is significantly upregulated [27]. This indicates that the autophagosome formation is normal, and that the occurrence of incomplete autophagy is due to the inhibition of autophagy flux. Significantly reduced autophagy flux can further inhibit the fusion of autophagosomes and lysosomes, leading to the accumulation of autophagosomes [70].

**Figure 1:**
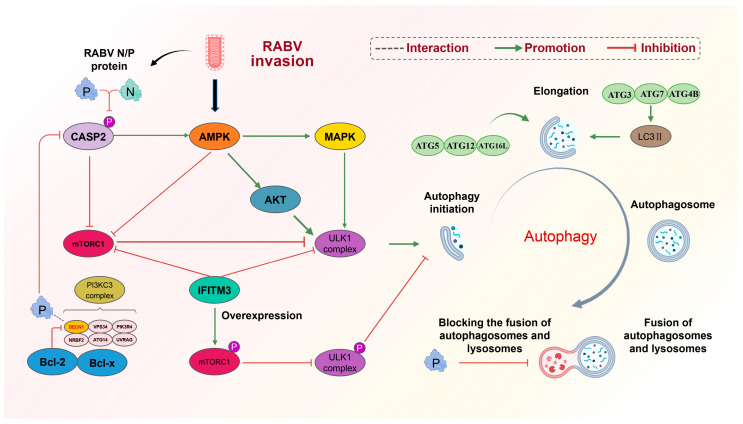
Mechanism of RABV-induced incomplete autophagy. (1) RABV activates the initiation of autophagy. RABV activates the AMPK signaling pathway upon invasion. On the one hand, activated AMPK inhibits mTORC1, thus blocking the inhibition of mTORC1 on the ULK1 complex. On the other hand, AMPK can positively regulate two factors, AKT and MAPK, to activate the ULK1 complex and prompt the initiation of autophagy. In addition, the P protein of rabies virus binds to Beclin1 in the PI3KC3 complex and reduces the phosphorylation of CASP2, which not only positively regulates the AMPK signaling pathway but also negatively regulates the mTORC1 signaling pathway, which activates the initiation of autophagy. (2) RABV prevents the fusion of autophagosome and lysosome: the P protein of RABV binds to Beclin1, wraps immature autophagosomes, inhibits the fusion of autophagosome and lysosome, and blocks the degradation of autophagosomes. (3) IFIM3 was able to directly inhibit the ULK1 complex and promote mTORC1 phosphorylation, indirectly inhibiting the ULK1 complex. Blocked the initiation of autophagy induced by RABV invasion.

The reported mechanism of RABV-induced autophagy is shown in Figure 1. Currently there is no clue as to why autophagy, as a defensive measure of the body, is conducive to RABV replication, nor to what the mechanism is by which autophagy promotes RABV replication. In addition, assuming that incomplete autophagy hinders the degradation of the virus by inhibiting the fusion of autophagosomes and lysosomes, it is still unclear what components are beneficial to the replication of RABV. To address these questions, further research is needed. In addition, how autophagy affects the pathogenicity of rabies virus has not been studied, and this is likely to be a new direction for the exploration of rabies virus.

## 3. Overview of Apoptosis

The term apoptosis was first introduced by Kerr et al. in 1972 to describe a morphologically distinct type of cell death [83]. Apoptotic cells undergo morphological changes including chromatin condensation, nuclear fragmentation, and cell shrinkage. Apoptosis is commonly referred to as programmed cell death (type I cell death), which is highly conserved in multicellular organisms and is under genetic control [84]. The mechanism of apoptosis is complex and involves pathways of multiple protein families. To date, apoptosis has been reported to be activated mainly through two pathways [31].

### 3.1. Mechanisms of Apoptosis

It is known that apoptosis is regulated by the intrinsic and extrinsic pathways [85]. Extrinsic apoptosis is mainly caused by the binding of external signals such as ligands to death receptors [86]. Common death ligands and receptors include tumor necrosis factor-alpha (TNF-α) and TNF receptor 1 (TNFR1), Fas ligand (FasL or CD95L or CD178) and Fas, TNF-like ligand 1A (TL1A) and death receptor 3 (DR3), TNF-related apoptosis-inducing ligand (TRAIL) and death receptor 4 (DR4), etc. [87,88]. Once these ligands bind to the corresponding death receptors, the specific death receptor can interact with the death domain (DD) of the adaptor proteins. Two adaptor proteins are TNF receptor-associated death domain protein (TRADD) and Fas-associated death domain protein (FADD) [89,90]. In these adapter proteins, there is another protein interaction domain called the death effector domain (DED) [91]. The adapter protein acts as a bridge, connecting on one side to the death receptor through the DD and on the other side recruiting and activating the caspase precursor through the DED, initiating the extrinsic apoptosis pathway. Studies have shown that caspase-8 and caspase-10 play a key role as “initiators” being the most upstream regulatory genes of the death receptor apoptosis pathway [92,93], activating downstream “executors” caspase-3, caspase-6, and caspase-7. Similarly, in the mitochondrial pathway (intrinsic apoptosis), caspase-9 acts as an “initiator” to activate the downstream “executors” caspase-3, caspase-6, and caspase-7. When the adaptor protein binds to the death receptor and the “initiators” caspases, a death-inducing signaling complex (DISC) is formed. This complex activates caspase-8 and caspase-10 through cleavage, and apoptosis is successfully initiated.

In extrinsic apoptosis, the initiation induced by the death ligand can be inhibited by FLICE-like inhibitory protein (FLIP). FLIP is a homologous protein of caspase-8 and has a similar DED, which can competitively bind to the death receptor [94]. In the DSIC containing FLIP, the enzymatic activity is low, so it cannot activate the casepase-8 zymogen and transmit death signals. Hence, FLIP is usually considered a negative regulator of apoptosis.

Intrinsic apoptosis is mainly mediated by mitochondrial proteins [95]. Typically, in response to various stimuli, the combined action of pro-apoptotic and anti-apoptotic proteins in the Bcl-2 family is shifted towards apoptosis, which results in a decrease in the mitochondrial membrane potential, causing cytochrome c release into the cytoplasm [85,96]. Cytochrome c can then bind to the apoptotic protease activating factor 1 (Apaf-1). Apaf-1, in the presence of dATP/ATP, undergoes oligomerization and conformational changes and can further bind to caspase 9, forming a pro-apoptotic complex named apoptosome, where caspase-9 is activated. Activated caspase-9 can further cleave and activate caspase 3, leading to apoptosis [97]. Additionally, mitochondria can also release endonuclease G (Endo G) and apoptosis-inducing factor (AIF), which can promote apoptosis by inducing chromatin aggregation and nuclear DNA breakage without relying on caspases [98].

Intrinsic apoptosis can be inhibited by the inhibitor of apoptosis protein (IAP) family [99]. The IAP family is conservative, including X-linked IAP (XIAP), cellular IAP1 (CIAP1), cellular IAP2 (CIAP2), neuronal apoptosis inhibitory protein (NAIP), survivin, etc. [100]. They all contain at least one BIR (baculovirus IAP repeat) domain, which can specifically bind to the target caspase to exert anti-apoptotic effects. However, DIABLO (also named SMAC, second mitochondria-derived activator of caspases) that is released by mitochondria can bind to the BIR domain of IAP, thereby competitively preventing IAP from binding to caspase and blocking its anti-apoptotic effect.

Of note, the extrinsic and intrinsic apoptosis pathways are not independent of each other [86]. The Bid protein in the Bcl-2 family is one of the substrates of caspase-8. Once the Bid protein is cleaved, the truncated Bid (tBid) can insert into the mitochondrial membrane, causing mitochondrial outer membrane permeabilization (MOMP), cytochrome c release, and apoptosome formation. Therefore, the Bid protein connects the intrinsic and extrinsic pathways [101].

### 3.2. Complex Interactions between Apoptosis and Viruses

Like autophagy, apoptosis and viruses also have complex interactions [102]. On the one hand, apoptosis of host cells invaded by viruses can serve as a defense mechanism of the body, clearing viruses or inhibiting virus replication by “suicide” and further preventing the virus from invading nearby healthy cells. Correspondingly, many viruses weaken the host immune response and promote their own proliferation by evading, hindering, or disrupting apoptosis of host cells [103]. For example, the V protein of Newcastle disease virus can inhibit apoptosis, thereby promoting its own replication [104,105]. On the other hand, due to the evolution and variation of some viruses, apoptosis during their infection can also enhance virus replication. The N protein of Caprine parainfluenza virus type 3 (CPIV3) activates apoptosis through both intrinsic and extrinsic pathways, and this pro-apoptotic mechanism also enhances CPIV3 replication [106]. It is worth noting that some viruses can promote their survival and reproduction by regulating apoptosis of host cells at different stages. Influenza A virus inhibits apoptosis by upregulating the PI3K-AKT pathway in the early stages of replication, while it promotes apoptosis by upregulating the p53 expression in the later stages of virus replication. Inhibiting apoptosis allows for the virus to have ample time to replicate and mature; promoting apoptosis helps release the progeny virus and expand the range of infection [107]. In short, viruses break the balance between pro-apoptotic and anti-apoptotic proteins, thereby controlling apoptosis to achieve their goal of escaping and replication.

### 3.3. RABV and Cell Apoptosis

It is currently known that RABV can cause apoptosis in infected cells [108], and this apoptotic action often inhibits RABV replication. Proteins crucial for apoptosis are generally pointed to G, M, and P. However, the detailed mechanisms by which RABV induces apoptosis are not fully understood.

#### 3.3.1. RABV G-Induced Apoptosis

Studies have demonstrated that the expression level of the G protein of RABV is a key factor in inducing apoptosis [109,110]. It has been described that infection with a double G recombinant RABV significantly increases the G protein level in NA cells, which is accompanied by an increase in cell apoptosis and an elevated immune response [111]. In addition, the accumulation of the viral G protein can also induce apoptosis in the cells infected with the attenuated strain ERA. Interestingly, compared to the pathogenic strain CVS, the attenuated strain ERA has a higher G protein accumulation and causes more T lymphocyte apoptosis [112]. Thus, the apoptosis induced by RABV is negatively correlated with the virulence of the virus and positively correlated with the expression level of the G protein. Different RABV strains have significantly different abilities in inducing cell apoptosis, which is possibly associated with the different amino acid sequences and maturation pathways of the G protein [111]. To explore the pathogenic mechanism of RABV, Li et al. made recombinant RABV SAD strains by mutating G (K83→R and P367→S) in the wild type RABV SAD strain. They found that mutation of G (K83R) of the RABV SAD strain significantly reduced the virulence, and the increased expression of the mutant G protein induced more apoptosis in infected cells [109]. Babault et al. demonstrated that the C-terminal peptide of the RABV G protein could target the PDZ domain of PTPN4 (protein tyrosine phosphatase non-receptor type 4), promoting glioblastoma cell apoptosis [110]. It is known that PTPN4 can inhibit apoptosis, promoting cell survival. Moreover, as the RABV G protein is synthesized in the cytoplasm and exerts its pathogenic role by anchoring in the cytoplasmic membrane [113], it is speculated that the accumulation of the G protein can cause oxidative stress in cells, inducing apoptosis by targeting the NF-κB signaling pathway [114]. Nevertheless, this viewpoint remains to be validated.

G protein-coupled receptors (GPCRs) are a superfamily of cell membrane-located receptors, recognizing various external stimuli and activating intracellular G proteins for signal transduction [115]. G protein-coupled receptor 17 (GPR17) is one member of the GPCR family, which is primarily expressed in the central nervous system and plays a vital role in the repair and differentiation process of damaged neurons [116]. Liu et al. found that during RABV infection, overexpressing or activating GPR17 reduces RABV replication, while silencing or deactivating GPR17 increases RABV replication in N2a cells [117]. They also observed that overexpression of GPR17 enhances RABV-induced apoptosis, which is associated with upregulation of pro-apoptotic gene BAK. Interestingly, knockdown of BAK attenuates GPR17-induced inhibition of RABV replication [58]. These findings suggest that GPR17 inhibits RABV replication by promoting BAK-mediated intrinsic apoptosis.

#### 3.3.2. RABV M-Induced Intrinsic Apoptosis

It has been shown that the RABV CVS strain can induce apoptosis of N2a cells at 72 hpi [118]. Upon examining apoptosis-related genes, RABV CVS was found to significantly activate caspase-3, caspase-9, and caspase-8. Blocking the activation of caspase-9 rather than caspase-8 reduces the cleavage of caspase-3 and apoptosis [119], indicating that the apoptosis induced by CVS mainly comes from the mitochondrial pathway. In addition, overexpression of the M protein alone can cause a decrease in mitochondrial membrane potential in a time-dependent manner [120,121]. Furthermore, the M protein of CVS co-localizes with the mitochondrial outer membrane translocase 20 (TOMM20) [122]. Apoptosis via the mitochondrial pathway is mainly regulated by the Bcl-2 family. CVS inhibits apoptosis by downregulating the expression of pro-apoptotic gene BAX in the early stages of infection, but triggers apoptosis in the later stages of infection by increasing the release of cytochrome c and upregulating the expression of AIF [123,124]. This results in caspase-dependent and -independent apoptosis of N2a cells. Moreover, the codon de-optimized M protein of RABV can induce a higher level of cell apoptosis [124]. It has also been described that chimeric recombinant viruses carrying the wild-type RABV *M* gene can induce the conversion of LC3-I to LC3-II in SK and NA cells, which reduces cell apoptosis [27]. Additionally, the *M* gene of GD-SH-01 might also cooperatively induce autophagy [27,125].

#### 3.3.3. RABV P-Induced Apoptosis

Previous studies have shown that the G and M proteins play a crucial role in apoptosis induced by RABV attenuated strains, but the P protein may play an important role in apoptosis induced by the virulent strain GD-SH-01 [122]. Recently, it has been shown that infection of GD-SH-01 or the HEP-Flury recombinant strain carrying GD-SH-01 *P* gene (rHEP-shP) results in the downregulation of Bcl-2, a decrease in mitochondrial membrane potential, an increase in mitochondrial cytochrome c release, and the activation of caspase-9 and caspase-3 [27,126]. The results confirm that the P protein of RABV can induce intrinsic apoptosis in the virulent strain GD-SH-01. However, expression of P protein alone hardly induces apoptosis [114,127], suggesting that the P protein needs the assistance of other components in the process of inducing apoptosis. Further research is required to unveil these components.

G protein induces mitochondrial apoptosis by upregulating the expression of pro-apoptotic genes BAK and BAX. Like M protein, it causes apoptosis through caspase-dependent and non-caspase-dependent pathways. P protein does not regulate apoptosis independently but causes apoptosis indirectly by increasing the accumulation and expression of G protein.

The ability of RABV to induce apoptosis is not only related to the replication level of the virus, but also related to the proportion and location of the related genes. To study the effect of P protein rearrangement on apoptosis, Mei et al. rearranged the P protein position in RABV HEP-Flury [128]. They found that compared with the wild-type strain at position 2, the recombinant genes *P1*, *P3*, and *P4* can cause apoptosis, but the apoptosis rate caused by *P1* and *P3* is lower than that of the control group HEP-Flury, and the ability of *P4* to induce apoptosis is comparable to that of HEP-Flury. The reason behind this is that the rearrangement of *P4* makes the *G* gene closer to the promoter, increasing the expression of G protein [128]. Therefore, in the attenuated HEP-Flury, the P protein does not directly induce apoptosis, but indirectly upregulates the expression level of the G protein to promote apoptosis.

**Figure 2:**
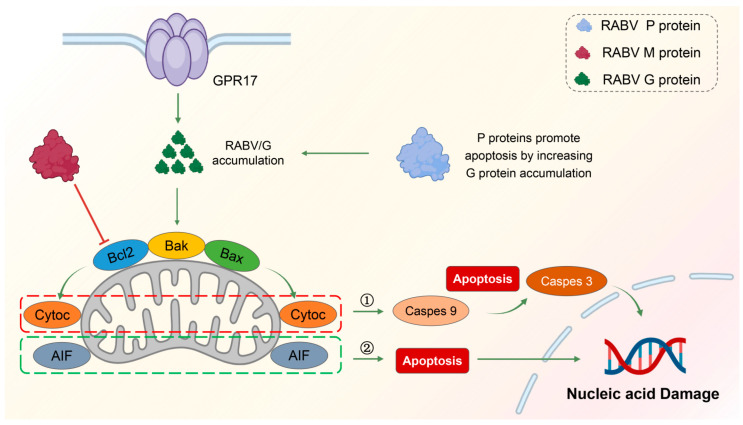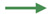: promotion 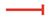: inhabition. Mechanism of RABV-induced mitochondrial apoptosis. RABV is able to cause mitochondrial apoptosis in late replication and is regulated by the *BCL-2* family of genes. The three proteins of RABV, M, G, and P are closely related to apoptosis. The M protein inhibits the anti-apoptotic gene *Bcl2*, causing mitochondrial apoptosis and the release of cytochrome C and AIF factors. Caspase-dependent pathway (➀): released cytochrome C activates caspase-9, which further activates the downstream caspase-3, causing apoptosis. Non-caspase-dependent pathway (➁): released AIF can directly cause apoptosis.

In general, the P protein of RABV contributes to the induction of apoptosis, but the P protein alone is not enough to induce apoptosis, and the mechanism of action of the P protein will be different due to the difference between the strains and the host cells. However, P protein is still a key molecule for studying RABV-triggered apoptosis. Figure 2 summarizes the mechanisms of apoptosis regulated by the G, M, and P proteins of RABV.

## 4. Interplay between Autophagy and Apoptosis in RABV

Due to the interactions between autophagy and apoptosis-related proteins, and the involvement of common transcriptional regulators and signaling pathways, such as p53, NF-κB, and PI3K/AKT, a foundation is established for the crosstalk between autophagy and apoptosis [129]. During the infection process of RABV, it has been confirmed that both autophagy and apoptosis can be induced in host cells. Studies have found a certain connection between autophagy and apoptosis, but the pathways of crosstalk between autophagy and apoptosis are only beginning to be recognized. At low exposure levels, host cells first maintain cellular homeostasis by autophagy to suppress apoptosis; when the exposure level reaches a certain threshold, autophagy alone is insufficient to protect cells. At this point, autophagy further promotes apoptosis, synergistically inducing cell death, to minimize damage to the body [130].

### 4.1. Inhibition of Autophagic Flux and Promotion of Apoptosis by M Protein

Peng et al. found that RABV GD-SH-01 can simultaneously trigger both cell apoptosis and autophagy [128]. Further exploring the relationship between the two cellular events revealed that the M protein of GD-SH-01 links autophagy and apoptosis. The *M* gene is the primary gene promoting RABV-induced autophagy. When the *M* gene of the attenuated HEP-Flury strain is replaced with the *M* gene of the pathogenic GD-SH-01 strain, the apoptosis rates increase in both SK and NA cells [27,114]. Moreover, in M-induced autophagy, activated caspase-3 can inhibit the autophagy flux, thereby inducing apoptosis [131]. Thus, the M protein of GD-SH-01 promotes the occurrence of both autophagy and apoptosis, with autophagy preceding apoptosis, possibly as a protective mechanism against cell apoptosis.

### 4.2. Enhanced Autophagy Flux and Inhibition of Apoptosis by Bif-1

Bax interacting factor-1 (Bif-1) is a multifunctional protein, involved not only in the process of cell apoptosis but also closely related to the formation of autophagosomes and the regulation of mitochondrial morphology [132]. Studies have shown that Bif-1 binds and activates BAX to promote apoptosis [117]. Bif-1 also interacts with UVRAG and BECN1, regulating the activity of the class III PI3K complex and participating in the formation of autophagosomes [133,134]. Three subtypes of Bif-1 (Bif-1b/c/e) are expressed in neuronal cells, where Bif-1c regulates the autophagy flux, eliminating the accumulation of autophagosomes to facilitate the autophagy process and inhibit RABV replication [135]. Neurons selectively express longer subtypes of Bif-1, inhibiting cell apoptosis and promoting neuronal survival for neuroprotection [136]. Bif-1c participates in RABV-induced autophagy and inhibits cell apoptosis, aiming to protect neuronal cells and hinder RABV replication [135].

## 5. Conclusions

RABV can stimulate autophagy through various means. For instance, the N/P proteins act early in the onset of autophagy, downregulating CASP2, inhibiting mTOR, and phosphorylating AMPK and MAPK to induce the initiation of autophagy. For the mid-late stages of autophagy, including the nucleation of autophagosomes, the extension of the bilayer, and the fusion process of autophagosomes and lysosomes, RABV mainly induces incomplete autophagy by binding the P protein to BECN1. The P5 protein binds directly to the N-terminal cyclic structure of BECN1, with the P5 protein forming a cyclic structure wrapping around immature autophagic vesicles, preventing the fusion of autophagosomes with lysosomes. In short, RABV induces and utilizes autophagy through different pathways to assist its replication. Conversely, the host has corresponding measures to suppress the virus replication. IFITM3 inhibits RABV replication by suppressing mTORC1/ULK1-dependent autophagy. Moreover, during the invasion of RABV, cell apoptosis is activated through diverse pathways. The expression level of RABV G protein is positively correlated with the level of apoptosis. That is, during the replication process of RABV, the higher the replication level, the more the G protein content. At this time point, apoptosis serves as a defense measure, inducing host cells to “commit suicide” to suppress virus replication and further spread. Both M and P proteins can target mitochondria, inducing intrinsic apoptosis through caspase-dependent and -independent pathways. The optimized codon of the M protein can induce a higher level of cell apoptosis. However, the P protein alone is insufficient to induce cell apoptosis but indirectly increases the expression level of the G protein to promote apoptosis.

Both P and M proteins participate in the processes of autophagy and apoptosis, indicating that there is a crosstalk between autophagy and apoptosis during the RABV infection process (Table 1). Autophagy usually occurs before apoptosis, and various genes regulate apoptosis by adjusting the autophagy flux. There are two scenarios: the M protein of GD-SH-01 induces cell apoptosis by activating caspase-3 to inhibit autophagy flux; neuronal cells selectively express Bif-1c to eliminate the accumulation of autophagosomes and make autophagy flow smoothly to inhibit cell apoptosis. Regardless of the scenario, they play an inhibitory role in RABV replication. This indicates that cell apoptosis plays a protective role for the organism, although the detailed mechanism remains to be determined. The complex regulatory network may also have significantly different or even opposite effects due to the differences in host cells and RABV strains. Further elucidating the regulatory mechanisms of RABV proteins in autophagy and apoptosis as well as the key nodes between the two cellular events will provide new insights into pathogenesis of rabies and help develop new therapies for the treatment of rabies [137].

## Figures and Tables

**Table 1:** The role of RABV proteins involved in autophagy and apoptosis and their effects on viral replication.

Proteins	G	M	P	N	Effect on RABV Replication	References
Autophagy		Induce autophagy	1. Decrease CASP2 2. Activate mTOR 3. Interact with BECN1 4. Inhibit the fusion of autophagolysosomes	1. Decrease CASP2 2. Activate mTOR	Promotion	[27,58,61,63,64,65,66,67,68,69]
Apoptosis	1. Expression level proportional to the level of apoptosis 2. Target PTPN4 via C-terminal PDZ domain 3. Upregulate pro-apoptotic gene BAK	1. Target mitochondria 2. Upregulate AIF and increase cytochrome c release 3. Co-localize with TOMM20	1. Involved in intrinsic apoptosis 2. Downregulate of anti-apoptotic gene Bcl-2 3. Assist G protein in inducing apoptosis		Inhibition	[27,108,109,110,112,115,118,119,122,126,127,128]
Crosstalk between autophagy and apoptosis		Inhibition of autophagic flux by caspase-3 induces apoptosis	Involved in autophagy and apoptosis			[27,114,117,128,131,132,133,134,135,136]
